# Phytochemical Composition, Antioxidant, Antibacterial, and Enzyme Inhibitory Activities of Various Organic Extracts from *Apocynum hendersonii* (Hook.f.) Woodson

**DOI:** 10.3390/plants11151964

**Published:** 2022-07-28

**Authors:** Aminu Shehu Abubakar, Xiaoyu Huang, Ziggiju Mesenbet Birhanie, Gang Gao, Xinkang Feng, Chunming Yu, Ping Chen, Jikang Chen, Kunmei Chen, Xiaofei Wang, Aiguo Zhu

**Affiliations:** 1 Institute of Bast Fiber Crops, Chinese Academy of Agricultural Sciences, Changsha 410205, China; aashehu.agr@buk.edu.ng (A.S.A.); huangxiaoyu0314@163.com (X.H.); zegje23@gmail.com (Z.M.B.); gaogang@caas.cn (G.G.); fengxk.pangry@foxmail.com (X.F.); yuchunming@caas.cn (C.Y.); chenping02@caas.cn (P.C.); chenjikang@caas.cn (J.C.); chenkunmei@caas.cn (K.C.); wangxiaofei@caas.cn (X.W.); 2 Department of Agronomy, Bayero University Kano, Kano PMB 3011, Nigeria

**Keywords:** *Apocynum hendersonii*, flavonoids, phenolics, tannins, polysaccharides, antioxidant, antimicrobial, tyrosinase, acetylcholinesterase

## Abstract

*Apocynum hendersonii is* a traditional medicinal plant used primarily as tea. It has a potential health benefit from its rich bioactive substances. This study investigated the reactivity of solvents of different polarities (ethanol, ethyl acetate, n-hexane, methanol, and water) extracts of the *A. hendersonii* leaf. The phytochemical composition of the extracts was evaluated using a Fourier Transform Infrared spectrophotometer (FT-IR), Gas Chromatography-Mass Spectrometry (GC-MS), UHPLC-MS, and Higher Performance Liquid Chromatography (HPLC). The result revealed the presence of medicinally important bioactive constituents, including phenols, flavonoids, and polysaccharides. Methanol extracts exhibited the highest flavonoid contents (20.11 ± 0.85 mg QE/g DW) and the second-highest in terms of phenolic (9.25 ± 0.03 mg GAE/g DW) and polysaccharide (119.66 ± 2.65 mg GE/g DW). It also had the highest antioxidant capacity with 60.30 ± 0.52% and 4.60 ± 0.02 µmol Fe^2+^ per g DW based on a 2,2-diphenyl-1-picrylhydrazyl (DPPH) radical scavenging assay and ferric reducing antioxidant power (FRAP), respectively. Ethanol extract displayed the maximum antibacterial action against Gram-negative and Gram-positive bacteria and the highest inhibition activity against the enzymes tyrosinase and acetylcholinesterase, followed by methanol extract. The principal component analysis revealed a positive correlation between the constituents, bioactivities, and extracts. The overall result showed *A. hendersonii* as a rich natural source of antimicrobial and antioxidant bioactive compounds and may be used for future applications in pharmaceuticals and food industries.

## 1. Introduction

Plants are a major and rich source of biomolecules that have multifunctional activities and low drug resistance potential, eliciting few side effects [1]. Bioactive plant materials, such as flavonoids, phenols, polysaccharides, and terpenoids, have tremendous biological applications and have been exploited as medicine, cosmetics, and food additives [2]. Polyphenols and polysaccharides have been reported to have exhibited therapeutic action against oxidative stress scavenging on free radicals [3,4]. Free radicals are generated in living cells as a product of metabolism or due to endogenous and environmental factors [4,5,6]. Oxidative stress leads to diseases such as inflammation, diabetes, and cancer [6,7]. Synthetic antioxidants, such as butylhydroxyanisole and butylhydroxytoluene, are carcinogenic and lead to liver impairment and atherosclerosis [4,8]. Plant-based antioxidants presented a better alternative. Many findings demonstrated a positive correlation between plant polyphenols’ bioactive substances and antioxidant activity, increasing global interest in exploiting plants for medicinal purposes [4]. Traditional uses of plants or those produced from their safety level and availability have been promoted by botanists. Moreover, numerous plants and their products have been reported and scientifically proven to have medicinal significance against many diseases, such as cancer, Alzheimer’s, diabetes, fever, hepatopathy, etc. [9,10]. Due to the increasing demand for bioactive compound-rich plants, the limited number of available wild plants, and prevent endangering of target plant species, several other plants are being investigated for possible medicinal potentials [4,6]. *Apocynum hendersonii* is one of the best alternatives with significant therapeutic potential.

*Apocynum hendersonii* (Hook.f.) Woodson, also known as *A. pictum* (Schrenk) Baillon, belongs to the *Apocynaceae* family and is used as traditional herbal tea in China and Japan due to its rich quercetin content [11]. It is considered a substitute [12] or complementary to its closely related species—*A. venetum*, which exhibits broad medicinal functions [13]. The two *Apocynum* species are often confused together. However, *A. hendersonii* is distinguished by its comparably narrow leaves and conspicuous white flower, unlike *A. venetum*, which has a red-colored flower and broader leaves. Both species however have the same geographical distribution [14,15]. Several types of research papers on metabolomics, transcriptomics, genomics, and bioactivities were conducted mainly on *A. venetum* with only a few on the *A. hendersonii*. Extract of *A. hendersonii* flower has been reported to have enhanced adipogenesis of 3T3 L1 cells [12]. Metabolomic analysis of *A. hendersonii* showed a substantial amount of flavonoids in the plant [16]. Flavonoids are the most diverse and most common forms of phenolic compounds with very effective antioxidants capacity by chelating elements involved in the free radical formation or scavenging reactive oxidative species [13,15,17].

*A. hendersonii,* in addition to flavonoids or polyphenols, may contain polysaccharides, as *A. venetum* was recently reported to contain the bioactive substance [18]. Polysaccharides are macromolecules of biological importance with anti-inflammatory and immunomodulatory properties [19]. Studies have also indicated their significance in preventing oxidative damage and as dietary free radical scavenging molecules [20]. Polysaccharides from corn silk have exhibited antifatigue, antihepatoma, antiobesity, and antioxidant activity [21].

Medicinal properties, including antioxidants and antimicrobials of plant and plant products, are associated with their bioactive constituents, mainly phenolic metabolites [22]. Phenolic compounds encompassed phenolic acids, polyphenols (condensed tannins), and flavonoids [23]. Techniques such as the Soxhlet extraction, maceration, subcritical aqueous extraction, supercritical fluid, and ultrasound-assisted extraction methods are available for recovering plants’ polyphenols. The extraction technique employed and the solvents used affect the yield and bioactive components’ property. Solvents of different polarities, such as methanol, ethanol, ethyl acetate, acetone, or aqueous forms, are commonly used [23,24,25]. Selection of suitable solvent is paramount and will enhance extraction efficiency [26,27]. Therefore, this study evaluated the reactivity of extracts obtained from different solvents of *A. hendersonii*. Total polyphenols comprising phenolics, flavonoids, and tannins, and total polysaccharide contents were determined. FT-IR was used to identify compound functional groups in the extracts, GC-MS and UHPLC to identify various bioactive constituents. HPLC analysis was conducted to quantify some of the active phenolic compounds in the different extracts. Antioxidant activity was determined using FRAP, DPPH, and 2,2- azino- bis (3- ethylbenzo thiazoline-6-sulphonic acid) (ABTS), and antibacterial activity was assayed against Gram-positive and Gram-negative bacteria. Enzyme inhibition activity was also tested against tyrosinase and acetylcholinesterase.

## 2. Results and Discussions

### 2.1. Polyphenol Contents

**Figure 1:**
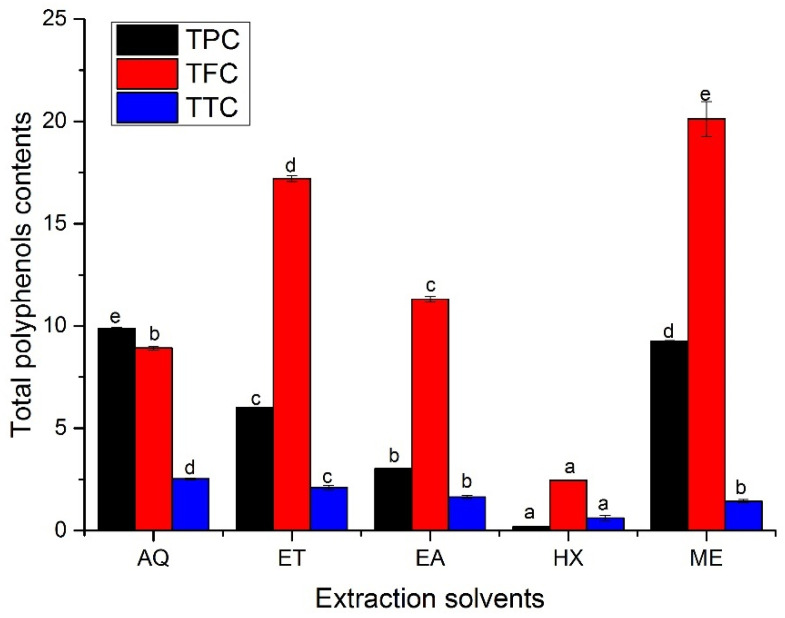
Total phenols, flavonoids, and tannin contents in the *A. hendersonii* leaf extracts obtained using various solvents. TPC: total phenol content (mg GAE/g DW); TFC: total flavonoid content (mg QE/g DW); TTC: total tannin content (mg CE/g DW); GAE: gallic acid equivalents; QE: quercetin equivalents; CE: Catechin equivalents. AQ: Water; ET: Ethanol; EA: Ethylacetate, HX: n-Hexane; ME: Methanol. Bars with different letters are statistically different (*p* < 0.05).

There was comparably higher significant variation (*p* < 0.05) in the total phenols (TPC), flavonoids (TFC), and tannin contents (TTC) in the various leaf extracts of *A. hendersonii*, as depicted in Figure 1. Total phenolic content was highest in the water extract (9.86 ± 0.05 mg GAE/g DW), followed by methanol leaf extract, which had phenolic content of 9.25 ± 0.03 mg GAE/g DW and ethanol extracts (6.00 ± 0.04 mg GAE/g DW). The lowest total phenolic content (0.19 ± 0.009 mg GAE/g DW) was obtained in n-hexane leaf extracts, which may not be unconnected to its non-polarity. The higher phenolic content obtained in water extract may have been attributed to higher sugar levels reacting with the Folin–Ciocalteu regent, leading to its overestimation [28], or due to the formation of some complex phenolic compound soluble in methanol or ethanol or from the presence of non-phenolic compounds such as carbohydrate or terpene in the aqueous extract [23]. It might also be due to the higher polarity of the water and comparably higher solubility of the phenols in water from its numerous hydroxyl group [5]. Methanol and ethanol or their aqueous forms are the most common solvents employed in the extraction of plant-based metabolites, and the choice of solvents has a significant role in yield and bioactive properties [23,24,25]. The methanol extract was reported to have had the highest phenolic contents in *Cupressus sempervirens* [29], *Ceropegia* spp. [30], and pomegranate peels [31], as obtained in this study.

The total flavonoid content in the leaf extracts showed higher significant diversity between the extraction solvents (*p* < 0.05) and comparably differed from that of total phenolic content, as shown in Figure 1. It ranged from 2.45 ± 0.00 to 20.11 ± 0.85 mg QE/g DW. Methanol leaf extract exhibited the highest total flavonoid content, followed by ethanol extract with 17.20 ± 0.14 mg QE/g DW. n-Hexane leaf extract was the least among the extracts. The low flavonoid content in the n-hexane extract was similar to *Limonium delicatulum* shoot extract [32], which could have been due to its non-polar characteristics [5]. In another study, the methanol extract was found to have the highest flavonoid content in *Meyna spinose* leaf extract, which was reflected in its higher antioxidant activity [4]. In contrast, the ethanol extract has had the highest flavonoid content in *Limnophila aromatica* extract compared to similar extraction solvents [23], which may not be unrelated to the flavonoid constituents in *A. hendersonii*.

The total tannin contents had different trends compared to the total phenolic and flavonoid contents. The highest (2.52 ± 0.05 mg CE/g DW) and lowest (0.61 ± 0.13 mg CE/g DW) tannin contents were obtained in water and n-hexane extracts, respectively. Ethanol and ethyl acetate extracts with 2.10 ± 0.10 and 1.64 ± 0.08 mg CE/g DW total tannins comparably have higher total tannin contents than methanol extract (1.43 ± 0.08 mg CE/g DW). Nevertheless, ethyl acetate and methanol extracts were statistically similar (*p* > 0.05). However, methanol extract was reported to have had the highest tannin content in *L. delicatulum* [32]. Previous studies have shown that polyphenols depend on several factors, such as species, genotypes, and environmental and edaphic factors [33]. In addition, the solvent polarity and degree of phenols polymerization and interaction play a significant role [32].

### 2.2. Polysaccharide Content

**Figure 2:**
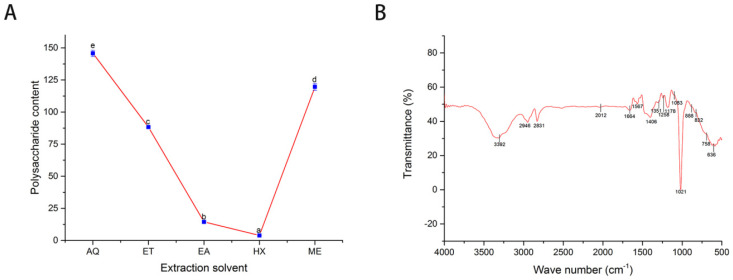
Polysaccharide contents (mg GE/g DW) of the various solvents (**A**) and FT-IR spectroscopy analysis of methanol extracts, showing characteristics peaks corresponding to the functional group of the constituents (**B**). GE: Glucose equivalent; AQ: Water; ET: Ethanol; EA: Ethyl acetate, HX: n-Hexane; ME: Methanol. The different letters in A indicate statistical differences (*p* < 0.05).

Polysaccharides are monosaccharides polymers joined by glycosidic bonds. They are reported to have antioxidant, antibacterial, antiviral, and antitumor properties, making them essential products in the pharmaceutical, food, and feed as well as cosmetic industries [34,35,36,37]. The polysaccharide content obtained from the various extracts is presented in Figure 2A. There was a significant difference (*p* < 0.05) among the extraction solvents, in the range of 3.89 ± 0.08 mg GE/g DW-145.74 ± 2.44 mg GE/g DW. Water-based leaf extract had the highest polysaccharide content, followed by methanol leaf extract (119.66 ± 2.65 mg GE/g DW) and ethanol leaf extract (88.33 ± 0.65 mg GE/g DW). The least polysaccharide content was obtained in n-hexane leaf extract. The extraction procedure was reported to affect the structural characteristics and antioxidant capacity of polysaccharides significantly. By extension, chemical composition and types of glycosides bond profoundly impact bioactivity [37]. Ethyl acetate or n-hexane may have distorted the polysaccharides configuration, leading to its low detection.

### 2.3. UV-Visible and Fourier Transform Infrared Spectrophotometers Analyses 

UV-Visible spectroscopy, in conjunction with FT-IR, is used to determine plant constituents’ properties [38]. The UV absorption spectra of the extracts (Figure S1) showed three characteristic peaks at 195, 231, and 271 nm, with the absorption at 195 being an expected peak of polysaccharides [39]. The absorption at 271 nm indicates the characteristic peak of flavonoids and their derivatives [38].

Fourier transform spectroscopy is crucial in structural determination of polysaccharides and reliable in obtaining information such as functional group and glycosidic bonds [20]. The result of FT-IR analysis is shown in Figure 2B. The absorption peak at 3392 cm^−1^ is the characteristics peak of OH groups, and peaks at 2946 and 2831 cm^−1^ correspond to C-H stretching; putting these together indicated the characteristics peak for polysaccharides [39]. A weak absorption peak at 2012 cm^−1^ showed the presence of aliphatic C-H stretching [20] and the one at 1664 cm^−1^ indicated a COO-carboxylate functional group [40]. The strong narrow peaks at 1567 and 1406 cm^−1^ represent the asymmetrical and symmetrical carboxylate anion stretching group (C=O) [34], and the few absorptions that fall between these two peaks might be due to the stretching vibration of N-H [20]. The peak absorption at 1258 cm^−1^ corresponded to sulfate vibration stretching of S-O [34], indicating possible sulfation of the *A. hendersonii* polysaccharide or other constituents. Sulfated polysaccharides have neuroprotective, anti-inflammatory, and immunomodulatory properties and protective properties against diabetic nephropathy [41]. Furthermore, the weak peak at 1351 cm^−1^ represents amine (C-N), and the strong peak at 1178 cm^−1^ indicates the presence of flavonoids [26,42].

The strong and weak absorbance at 1021 and 1083 cm^−1,^ respectively, might be due to the pyranose ring, which has characteristic peaks between 1000–1200 cm^−1^ [20,40]. The absorption peaks at 888, 832, and 758 cm^−1^ indicate the polysaccharides’ beta glycosidic, alpha structural configuration, and the asymmetrical ring vibration, respectively [20,39,40,43]. The glycosidic bond linkage, structural branches, composition, and degree of sulfation determined the bioactivity [34].

### 2.4. GC-MS Analysis

GC-MS is a valuable technique important in detecting, separating, and identifying constituents from a complex mixture [5]. The GC-MS analysis of the methanol leaf extract of *A. hendersonii* revealed about 20 compounds, as shown in Table 1, which were detected within the retention time of 5 to 55 min (Figure S2). Four sugar components, L-glucose, D-mannose, methylated glucose (3-O-Methyl-d-glucose), and β-Lactose, were detected at 0.87–5.39%. Mannose was the highest among the sugars and third highest among all the identified constituents after only fenpropathrin (8.66%) and palmitic acid (5.86%). Pyranone accounted for 4.76%. Mannose was similarly obtained as the main monosaccharide in the extract of seaweeds *Sarcodia ceylonensis* and *Ulva lactuca* [34]. Galactose was, however, reported as the main monosaccharide in *Poulownia fortunei* [19]. Polysaccharide extract from *U. lactuca* showed significant antioxidant capacity based on ABTS and hydroxyl radical scavenging assay [34].

### 2.5. UHPLC-MS Identification of Various Constituents and HPLC-Based Quantification of Some Essential Components in Various Extracts

To further identify more secondary metabolites constituents, including flavonoids, phenols, and sugars and support the GC-MS, UHPLC-MS analysis was carried out with the methanol extract. The compounds identified (Table 2, Figure S3) indicated the presence of medicinally important bioactive components. Many of them formed components of drugs for the treatment of many ailments. Rutin, for example, has antihypertensive, antiplatelet, antibacterial, antiviral, antiprotozoal, antitumor, and anti-inflammatory activity [44]. Epigallocatechin gallate and caffeic acid have exhibited antibacterial and antitubercular potencies [45,46]. Apigenin has antidiabetic activity and results in increased secretion of insulin. It is also neuroprotective, antiamyloidogenic, antidepressive, antitumor, and anti-inflammatory, and prevents insomnia [47]. Kaempferol is cardio-protective and neuroprotective and exhibits therapeutic properties against obesity, asthma, cancer, and diabetes [48,49]. Quercetin exhibits similar prophylactic properties as rutin and apigenin. Recently, in silico and in vitro studies reported that the flavonoid can interfere with various stages of coronavirus entry and replication [50].

HPLC analysis was performed to quantity some of the common flavonoid constituents in the various *A. hendersonii* leaf extracts. There were statistically significant differences (*p* < 0.05) across the different extracts. The flavonoids kaempferol, rutin, and apigenin were highest in the methanol leaf extract (Table 3). Ethanol leaf extracts had the highest quercetin level of 167.34 ± 2.59 µg/g, followed by methanol extracts with 113.49 ± 0.17 µg/g. n-Hexane leaf extracts had the lowest levels for all the flavonoid constituents quantified, followed by water-based extract. The low level of the flavonoids in water-based extract is in tandem that many flavonoids are not readily soluble in water, but they are in methanol and ethanol, which is directly reflected in the total flavonoids (Figure 1).

### 2.6. Antioxidant Capacity Assay

**Figure 3:**
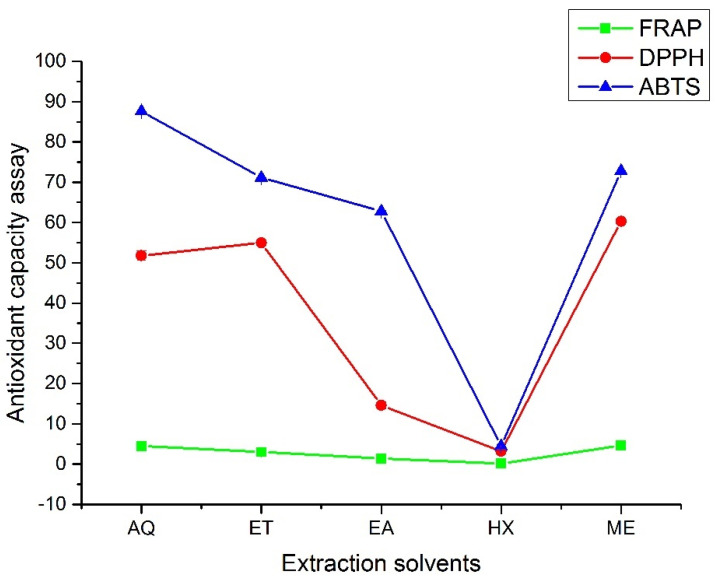
Antioxidant capacity assays of the *A. hendersonii* leaf extracts obtained using various solvents. FRAP: Ferric Reducing Antioxidant Potential (µmol Fe^2+^ per g DW); DPPH: 2,2-Diphenyl-1-picrylhydrazyl (%); ABTS: 2,2′-azino-bis (3-ethylbenzothiazoline-6-sulfonic acid (%); AQ: Water; ET: Ethanol; EA: Ethylacetate, HX: n-Hexane; ME: Methanol.

The result of the antioxidant activity of the *A. hendersonii* leaf extracts is presented in Figure 3. The values obtained ranged from 0.14 ± 0.00 to 4.60 ± 0.02 µmol Fe^2+^ per g DW, 3.19 ± 0.58 to 60.30 ± 0.52%, and 4.49 ± 0.42 to 87.64 ± 0.03% for FRAP, DPPH, and ABTS, respectively. Methanol leaf extract had the highest antioxidant activity, according to FRAP and DPPH. Ethanol leaf extract comes after methanol for DPPH with 54.96 ± 0.87% and third with 3.00 ± 0.01 µmol Fe^2+^ per g DW after methanol and water (4.50 ± 0.10 µmol Fe^2+^ per g DW) for FRAP. In both cases, n-hexane performed the least, reflecting its low polyphenols and polysaccharide contents. ABTS radical scavenging rate, however, slightly differed from the other two with results in the order Water > Methanol > Ethanol > Ethyl acetate > n-Hexane. Under acidic conditions, Fe^3+^-TPTZ is reduced to blue Fe^2+^-TPTZ, with the color changes reflecting the antioxidant capacity of the reacting substance. For ABTS, the blue color solution fades in the presence of antioxidants. DPPH reacts with antioxidants by pairing off an electron, resulting in the solution’s discoloration. The scavenging activity is reflected by the intensity of the discoloration and is directly dependent on electrons taken up [4]. The result obtained for FRAP is in line with what was obtained in *Ceropegia* spp., where methanol extracts gave the best radical scavenging activity [30]. It has been reported that extracts obtained from organic solvents have displayed more antioxidant power than water-based extracts obtained from many plant species. In a study on *Hibiscus cannabinus* leaf extracts, the water-based extract showed the lowest reducing power which was linked to the low solubility of the bioactive constituents in water [26] and this conforms with the result of FRAP and DPPH obtained in this study.

The *A. hendersonii* extract was shown to contain metabolites that cut across the phenols, flavonoids, and polysaccharides (Table 1 and Table 2), most of which possessed strong antioxidant activities. For example, Mannose-6-phosphate protects proteins in the dermis scaffold against oxidation and degradation [51]. Polysaccharides, comprising mannose, glucose and galactose from *Siraitia grosvenorii*, decreased the reactive oxygen species as well as the percentage of apoptotic and necrotic cells in H_2_O_2_ oxide injury PC12 cells [52], and these components were similarly identified in the *A. hendersonii* extract. Injection of L-fucose in rabbits resulted in increased production of vitamin C and promoted body defense against oxidative stress [53]. Quercetin is currently used in various pharmaceuticals preparation as an antioxidant and in the treatment of age-associated diseases [54]. Its mechanism of action involves the regulation of glutathione (GSH). Its phenyl ring hydroxyl groups bind to amino acid residues of the clinically important enzymes such as butyrylcholinesterase, resulting in a strong inhibitory effect [55]. Pretreatment with quercetin protected hippocampal CA1 pyramidal neurons from ischemic injury in gerbils by promoting the expression of endogenous antioxidants [56]. This further supported the antioxidant properties of the various extracts, as established in this study. Thus, the antioxidant capacity results obtained showed that *A. hendersonii* has excellent medicinal potential and would be a candidate for the pharmaceutical, food, and cosmetic industries.

### 2.7. Correlation Analysis of Bioactive Constituents and Antioxidant Capacity

Correlation analysis within the various constituents and with the FRAP, DPPH, and ABTS radical scavenging assays were computed to understand the strength of the relationship. FRAP (r = 0.99), DPPH (r = 0.92), and ABTS (r = 0.87) strongly correlated with total phenolic content as presented in Table 4. Flavonoid content showed a moderate correlation with FRAP (r = 0.677), DPPH (0.79), and ABTS (r = 0.67) compared to the total phenolic as similarly obtained in total tannins. Interestingly, polysaccharide contents correlated strongly with FRAP, DPPH, and ABTS with correlation coefficient values of 0.97, 0.92, and 0.79, respectively. A strong correlation was previously reported between polysaccharide content and total phenolic with antioxidant capacity [3].

Similarly, tannin content from leaves extract of *Ruta chalepensis* showed a higher positive correlation (0.95–0.99) with antioxidant capacity, and total flavonoids from the plant’s flower also correlated with free radical scavenging activity [57]. Flavonoid contents appeared to be the dominant form of polyphenols in the species, as similarly reported in guava [58] and *L. aromatica* [23]. Extraction solvents have significantly affected polyphenols levels, with methanol being the best solvent for flavonoids and phenolic than ethanol, ethyl acetate, or n-hexane. However, where condensed tannin content is the sole target, ethanol proved the best alternative, followed by methanol or ethyl acetate.

Antioxidant capacity showed a significant and positive correlation with polyphenols and polysaccharide contents, as most of these constituents are proven scavengers of free radicals. Antioxidants enhance the body’s defense system by shielding biomolecules against reactive oxygen species. The reactive oxygen species stressed the system, leading to diseases such as cardiovascular, cancer, liver cirrhosis, etc. Natural antioxidants, such as plant-based antioxidants, are always the best option compared to synthetic ones, which may have some side effects. Many studies have linked plants’ antioxidants and other medicinal properties or their extracts to their polyphenols, principally phenol and flavonoids [4]. The antioxidant property of *A. hendersonii* leaf extracts could have been due to multiple effects of all the bioactive components investigated, as each has a strong antioxidant capacity [3,23,37]. This confirms the species’ excellent nutraceuticals and pharmaceutical potential. This study might be the first to delve into assessing the polysaccharide content of this species. [4]

### 2.8. Antimicrobial Activity Assay

The assay result revealed the antagonistic effect of the ethanol, ethyl acetate, and methanol leaf extracts against the two bacterial strains, as given in Table 5. The three different extracts were statistically similar (*p* > 0.05) against the *Escherichia coli*, with the zone of inhibition of 10.33 ± 0.32, 9.15 ± 0.51, and 10.15 ± 0.60 mm, respectively. However, a significant difference (*p* < 0.05) was obtained against the *Staphylococcus aureus*, with ethanol leaf extract exhibiting the highest effect, having a 10.96 ± 0.15 mm zone of inhibition followed by methanol leaf extract with 8.90 ± 0.06 mm, as depicted in Figure S4. Water- and n-hexane-based extracts had no antibacterial effects against the two bacterial strains, which may not be unconnected to the significantly low flavonoid contents, as seen in Figure 1 and Table 3. The result is indicative of a positive correlation between flavonoid contents of the *A. hendersonii* extracts and antibacterial activity consistent with the literature [59].

The higher antibacterial effect of ethanol extract may have been due to the additional effect of the solvent, as its aqueous form is often used as sterilizing agent [60] and as seen in the negative control. Tannin, which has strong bactericidal activity [61], might have strengthened the antibacterial effect of the extract. It may have also been because bioactive components responsible for the antibacterial effect were readily soluble in the ethanol but not in the other solvents [62]. The antibacterial inhibition might have occurred due to extracts interacting with extracellular microbial enzymes, inhibiting substrates required for the bacterial growth and development or inhibiting metabolism via deprivation of oxidative phosphorylation [61]. Though the positive control displayed a higher effect against the bacterial strains with 12.15 ± 0.06 and 11.10 ± 0.13 mm zones of inhibition for *E. coli* and *S. aureus,* respectively, the plant extracts are still the best alternative because of the rapid emergence of resistance strains [63]. Ethanol leaf extract of *Hibiscus cannibinus* was reported to have imparted the highest antibacterial effect against *Bacillus cereus*, *Pseudomonas*, *Salmonella,* and *S. epidermis*; at the same time, methanol extract displayed the best effect against *B. subtilis* and *S. auerus* [26]. The differential antibacterial effects against the two different bacteria might be strain-dependent [57]. The overall result, thus, displayed great antibacterial potentials of the extracts and the plant, by extension.

### 2.9. Enzyme Inhibition Assay

Tyrosine inhibitors are being targeted as a treatment for Parkinson’s disease. Tyrosine partakes in neuromelanin formation in the human brain leading to dopamine neurotoxicity and neurodegeneration [64]. They also play a significant role in melanin biosynthesis by protecting the skin against ultraviolet light and reactive oxygen species [65]. There is a need for alternative drugs to stop these associated neurodegenerative diseases, with plants being an attractive source [64]. The tyrosinase inhibition assay showed great potential for the *A. hendersonii* in tyrosinase inhibition. There were, however, significant differences among the extracts, with ethanol extract exhibiting the highest inhibition percentage (62.30 ± 1.11%), followed by methanol (55.97 ± 0.89%) and water (32.10 ± 0.41) extracts. Hexane and ethyl acetate displayed the lowest inhibition percentage (Table 6). The higher variation obtained for the different extracts may be explained by their polyphenols contents, which are consistent with the result. Constituents with strong antioxidant activity tend to have good tyrosinase inhibition [26]. This may be the first report on the tyrosinase inhibition activity of the extracts, and as such, provides a basis and reason to explore many other clinically essential enzymes.

AChE inhibitors are also used to treat neurodegenerative diseases such as Alzheimer’s, prevalent in older adults. The available synthetic drugs used to treat such ailments are not without side effects and limited bioavailability [66]. In addition, such synthetic drugs do not stop the diseases but slow their progression via symptomatic treatment, which calls for better alternatives, with the plant being an attractive source [64]. The result obtained for AChE inhibition of the various *A. hendersonii* extracts followed similar trends with the tyrosinase inhibition activity, with ethanol extract displaying the highest effect (30.65 ± 0.98%) followed by methanol extract (28.60 ± 0.04%). Nevertheless, the two extracts were statistically similar (*p* < 0.05). The two results indicated that *A. hendersonii* is a potential candidate for inhibition of clinically important enzymes, and the variation in the extracts relates to the component bioactive substance.

### 2.10. Multivariate Analyses

**Figure 4:**
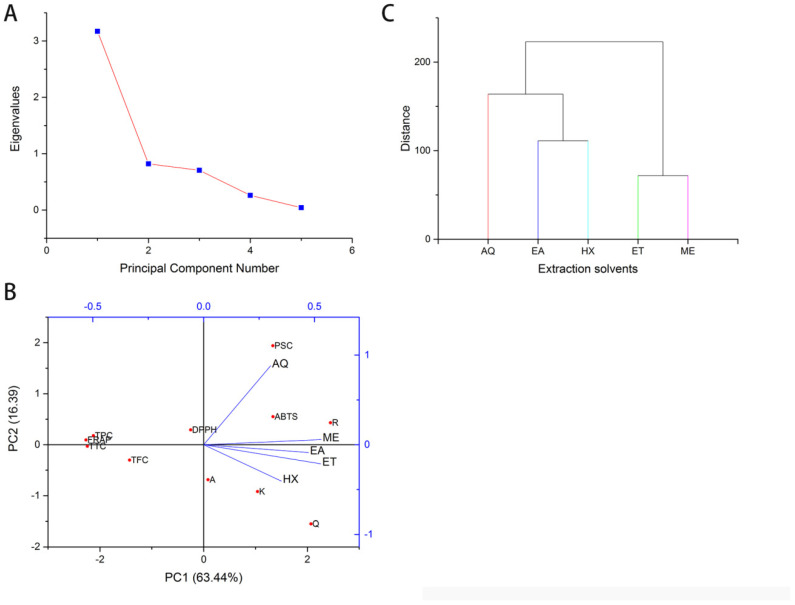
Principal component analysis and hierarchical cluster analysis of the various extracts, constituents, and bioactivities. (**A**) Eigenvalues of the variances. (**B**). Contribution of bioactive constituents to the principal components 1 and 2. (**C**). Dendogram of cluster analysis. FRAP: Ferric Reducing Antioxidant Potential; DPPH: 2,2-Diphenyl-1-picrylhydrazyl; ABTS: 2,2′-azino-bis (3-ethylbenzothiazoline-6-sulfonic acid (%); AQ: Water; ET: Ethanol; EA: Ethyl acetate, HX: n-Hexane; ME: Methanol; A: apigenin; R: rutin; Q: quercetin; K: kaempferol.

Multivariate analyses were carried out to understand further the overall variations obtained among the solvents’ reactivity. The univariate analysis revealed a tremendous variability in the bioactive constituents and bioactivities. Principle component analysis (PCA) and hierarchical cluster analysis (HCA) were computed to explain the correlation between the various components. PCA was obtained using the Kaiser Criterion, and the Eigenvalue is given in Figure 4A. TPC, TFC, TTC, the flavonoids apigenin, kaempferol, quercetin, rutin, and the FRAP, DPPH, and ABTS accounted for the variations in PC1 (63.44%). PC2 (16.39%) accounted for the variations in the PSC (Figure 4B). The considerable variability among the extraction solvents can be seen from the scatter plot, with methanol, ethanol, and ethyl acetate seeming very close. The result of HCA is presented in Figure 4C, which shows all the extracts clustering into five unique clusters in tandem with PCA proving that the extraction solvents have tremendously affected the bioactive constituents and activities. The result is similar to the findings on *Sphaeranthus indicus* [10]. Therefore, in selecting extraction solvents for plant bioactive components, the target constituents and their activities should be considered to preserve quality and maintain chemical structure [10].

## 3. Materials and Methods

### 3.1. Chemicals

Hexane and methanol were purchased from Sinopharm Chemical Reagent Co., Ltd., Shanghai, China and ethyl acetate and ethanol from Hunan Huihong Reagent Co., Ltd. (Hunan, China). Folin-Ciocalteu reagent and vanillin were purchased from Coolaber Science and Technology, Beijing, China. DPPH, ABTS, and T-AOC (FRAP), apigenin, (+)-catechin, gallic acid, glucose, kaempferol, quercetin, luteolin, naringenin, and rutin, were all purchased from Solarbio Life Sciences, Beijing, China. HPLC grade acetic acid and acetonitrile were purchased from Tianjin Kemiou Chemical Reagent Co., Ltd. (Tianjin, China).

### 3.2. Plant Material and Extraction Procedure

*A. hendersonii* leaf was obtained from the Xinjiang Western Centre of the Chinese Academy of Agricultural Sciences, Changji, Xinjiang province. The leaf was freeze-dried and pulverized using a mill grinder and sieved to a fine powder.

According to a previous report [3], five different solvents comprising ethanol, ethyl acetate, n-hexane, methanol, and water were used for the extraction process. The sample-to-solvent ratio was 1:10. The mixture was refluxed for 2 h at 85 °C, and the supernatant was collected and centrifuged for 10 min at 10,000 rpm. The extract was then evaporated to dryness at reduced pressure or lyophilized and stored at −20 °C for subsequent analysis.

### 3.3. Determination of Polyphenols Contents

Total phenolic content was determined using the Folin–Ciocalteau method [67] as reported by Medini et al. [32], with slight modifications. In total, 1.5 mL 20% Folin–Ciocalteu reagent was added to 0.2 mL extracts (10 mg/mL), mixed, and allowed to stay for 5 min at room temperature. Next, 4 mL 7% sodium carbonate (Na_2_CO_3_) and distilled water to a final volume of 10 mL were added, and the mixture was kept for 90 min in the dark at room temperature. Absorbance was then recorded at 760 nm using a spectrophotometer. The gallic acid standard curve (0.05–0.3 mg/mL, R^2^ = 0.99) was used to express the total phenolic as milligram gallic acid equivalent per gram sample dry weight (mg GAE/g DW).

Total flavonoid content was determined using Aluminum Chloride (AlCl_3_) calorimetric method as reported by Rguez et al. [29] with some modifications. In total, 0.2 mL extracts (10 mg/mL) were diluted with 4 mL distilled water followed by the addition of 0.3 mL 5% NaNO_2_ and kept for 5 min at room temperature. Then, 0.3 mL 10% AlCl_3_ was added, and the mixture was kept for another 6 min. Later, 2 mL 4% sodium hydroxide was added, and the final volume was filled up to 10 mL. Absorbance was measured at 510 nm using a spectrophotometer. The quercetin standard curve (0.025–0.25 mg/mL, R^2^ = 0.99) was used to express the total flavonoids as milligram quercetin equivalent per gram sample dry weight (mg QE/g DW).

Total condensed tannins (proanthocyanidins) were determined according to the procedure reported by Rguez et al. [29] with few modifications. Next, 0.1 mL extracts (10 mg/mL) were diluted with 1 mL distilled water, and 6 mL 4% vanillin-methanol (*w*/*v*) added, followed by 3 mL concentrated sulphuric acid. Absorbance was recorded at 500 nm using a spectrophotometer, and the (+)-catechin standard curve (0.05–0.3 mg/mL, R^2^ = 0.99) was used to compute the total condensed tannins as milligram catechin equivalent per gram dry weight (mg CE/g DW).

### 3.4. Determination of Polysaccharide Contents

The polysaccharide content was determined using phenol-sulfuric acid calorimetry according to the literature [68]. A total of 1 mL 5% (*w*/*v*) phenol was added to 2 mL of the diluted extracts, followed by 5 mL concentrated sulphuric acid, which was mixed and boiled for 15 min. It was allowed to cool down to room temperature, and absorbance was taken at 490 nm using a spectrophotometer. A d-glucose standard curve (0.05–0.3 mg/mL, R^2^ = 0.99) was used to express the polysaccharide content as milligram glucose equivalent per gram sample dry weight (mg GE/g DW).

### 3.5. UV-Visible and Fourier Transform Infrared Spectrophotometers Analyses 

The extract was diluted to a 1 mg/mL final concentration and used for UV spectra. The UV-visible spectrum was obtained from 190–400 nm using a UV-3600 Plus UV-VIS-NIR Spectrophotometer (Shimadzu, Japan).

The structural-functional group of compounds in the extract was obtained using IRSpirit Fourier Transform Infrared spectrophotometer (Shimadzu, Japan). A drop of the extract was placed on the diamond crystal to form a thin film. The spectral scanning was taken at the frequency range of 4000 to 400 cm^−1^ at 4 cm^−1^ at room temperature.

### 3.6. Gas Chromatography-Mass Spectrometry Analysis 

Bioactive constituents in the methanol extract of the *A. hendersonii* leaf were obtained using 7890A Agilent Technology Gas chromatography-mass spectrometry (GC-MS). The instrument was equipped with an HP-5ms capillary column (30 m × 0.25 mm × 0.25 μm) with helium (purity > 99.99%) as carrier gas. The flow rate was set at 1.2 mL/min, and the sample injection volume was 1 μL with 1:5 diversion ratios. The injection and detection temperatures were set at 250 °C and 280 °C, respectively. The initial temperature was maintained at 60 °C for 2 min, before it was increased to 280 °C at 5 °C /min and kept for 9 min. The ionization mode was 70 eV with a scanning range of 40–400 amu, a scanning rate of 3.99, and a 3 min solvent delay. The fragmentation pattern was compared to the NIST database, and percentages were computed from the chromatogram peak area.

### 3.7. UHPLC-MS Analysis

UHPLC-MS analysis was performed for the methanol extract using ACQUITY UHPLC coupled with Triple TOF 5600 systems in both positive and negative modes, as previously reported [69], and the details are given in the Supplementary. The tentative constituents were identified by METLIN metabolite and Human Metabolome databases.

### 3.8. HPLC-Based Quantitative Identification of Flavonoids Constituents

Higher performance liquid chromatography (HPLC) analysis was used to quantify some common flavonoid constituents in the different extracts. The extracts were filtered using a 0.45 µm filter into small vials for HPLC analysis. The analysis was performed on SHIMADZU LC-20A, column Shim-pack GIST C18 5 µm (4.6 I.D. × 250 mm) with the following conditions: flow rate 0.8 mL/min and 10 µL injection volume at 25 °C. The mobile phases were 0.04% acetic acid in water (mobile phase A) and 0.04% acetic acid in acetonitrile (mobile phase B). The column was initially developed isocratically with 5% solvent B, followed by a linear gradient from 5 to 95% for 20 min. It was then isocratically washed with 95% B for 2 min, followed by 95 to 5% linear gradient for 0.1 min. The column was then developed isocratically with 5% B for 5 min [70]. Chromatogram was obtained at 330 nm, and apigenin, kaempferol, naringenin, luteolin, quercetin, and rutin were used as flavonoid standards for calibration.

### 3.9. Antioxidant Capacity 

#### 3.9.1. 2,2-Diphenyl-1-picrylhydrazyl (DPPH) Radical Scavenging Assay

DPPH assay was conducted following the report of Liang et al. [15] with slight modification. A total of 0.5 mL of samples of various concentrations were mixed with 2 mL of 1 mM DPPH in an ethanol working solution and allowed to stay for 30 min at room temperature. Absorbance was obtained at 515 nm using a spectrophotometer, and radical scavenging capacity was expressed in the form of an inhibition percentage as follows:

Inhibition (%) = (A_b_ − A_e_)/A_b_) × 100 (1)

where A_b_ = the absorbance of the DPPH working solution without the extract and A_e_ = absorbance of the reaction mixture.

#### 3.9.2. 2,2′-Azino-bis (3-Ethylbenzothiazoline-6-sulphonic Acid) (ABTS) Radical Scavenging Capacity Assay

A radical scavenging capacity assay was conducted following the method reported by Shang et al. [37] with slight modifications. Different sample extract (0.1 mL) was mixed with 1 mL ABTS working solution, kept in the dark, and allowed to stay for 6 min. Absorbance was obtained at 405 nm. Vitamin C was used as a positive control, and all tests were repeated three times. Radical scavenging capacity was computed using the above equation (Equation (1)), with the absorbance of ABTS working solution as blank.

#### 3.9.3. Ferric Reducing Antioxidant Power (FRAP) Total Antioxidant Capacity Assay

In acidic conditions, the Fe^3+^-TPTZ (2,4,6-tris(2-pyridyl)-s-triazine) are reduced to Fe^2+^-TPTZ, resulting in a change of the colorless former mixture to blue, which reflects the antioxidant capacity of the sample used to bring about the color changes. A FRAP assay was conducted following the report of Yi et al. [68] with some modifications. The working solution was prepared by mixing 300 mM acetate buffer (pH 3.6), 10 mM TPTZ, and 20 mM Iron (III) chloride in a 7:1:1 ratio. Next, 900 µL of the working solution was mixed with 30 µL extracts followed by 90 µL distilled water to obtain a final volume of 1 mL. The mixture was thoroughly mixed and allowed to stay for 10 min at room temperature. Absorbance was measured at 593 nm against a blank that contained the working solution without the extract. FeSO_4_ standard curve (0.05–0.00156 µmol/mL, R^2^ = 0.99) was used and the results expressed as µmol Fe^2+^/g DW. The entire test was replicated three times.

### 3.10. Antimicrobial Activity Assay

The antimicrobial assay was carried out using the disc diffusion method according to previous reports [3] on Luria Bertani agar plates. Two bacterial strains, *Escherichia coli*, ATCC8739—a Gram-negative strain—and *Staphylococcus aureus*, ATCC6538—a Gram-positive strain—were used. The bacterial inoculums suspension of each (100 µL, 1 × 10^7^ CFU) was uniformly swabbed on the plates and 10 µL of each of the extracts (10 mg/mL), later loaded on an individual sterile disc (6 mm), allowed to rest for 30 min at room temperature, followed by incubation at 37 °C for 18 h. Ampicillin (100 µM) was used as positive control and respective solvents used in the extraction process were used as a negative control. The circular zone formed around the disc was considered a zone of inhibition due to the extract and was measured using a Vernier caliper. All tests were replicated three times.

### 3.11. Enzyme Inhibition Assay

Enzyme inhibition assay was carried out for all the extracts against acetylcholinesterase (AChE) and tyrosinase. AChE inhibition activity was determined as reported by Orhan et al. [66], and the result was expressed as a percentage using the equation:

$$EnzymeInhibition\left(\%\right)=\left(\frac{Ac-As}{Ac}\right)\times 100$$

where ‘Ac’ is the absorbance of the control without the sample and ‘As’ is the absorbance of the mixture containing the sample.

For tyrosinase inhibition activity, L-tyrosine (100 µL, 5 mM), sodium phosphate buffer (20 µL, 0.1 M, pH 6.8), and the sample (40 µL) were loaded onto a 96-well microplate. This was followed by adding tyrosinase (200 units/mL) and incubation at 37 °C for 20 min [71]. Absorbance was obtained at 450 nm using a microplate reader. A blank containing all the components except for the enzyme was also recorded and subtracted from the samples [72]. The tyrosinase inhibition was expressed as a percentage using the above equation.

### 3.12. Statistical Analysis

All the assays were repeated three times, and the experimental data obtained were expressed as mean ± standard error of the mean (n = 3). The means of the total polyphenols, polysaccharide contents, and the results of antioxidant capacity assay were subjected to one-way analysis of variance using GenStat 17th edition and means separated using the Student–Newman–Keul method. Correlation analysis was performed using Statistix analytical software version 8.3. Principal component and hierarchical cluster analyses were carried out using Origin Pro for further understanding the bioactive constituents’ variability and their activities.

## 4. Conclusions

This study showed all the different extracts obtained with the various solvents to have a substantive amount of polyphenols and significant antioxidant properties, except for n-hexane, which has a minimal amount of polyphenols and displayed the lowest antioxidant capacity. Polysaccharide content in n-hexane and ethyl acetate extracts was comparably smaller than that of other solvents, reflecting its non-polar nature and indicating that polar solvents might be the best option for recovering polysaccharides in *A. hendersonii*. Methanol gave a better result with its extracts based having the highest flavonoids and comparably higher phenolic and showed the best antioxidant capacity. A strong positive correlation was obtained between the antioxidant capacity assays and polyphenols and polysaccharide contents of the extracts indicating the total impacts of the constituents in conferring the medicinal significance of the *A. hendersonii*. Furthermore, HPLC analysis of some of the flavonoid constituents revealed apigenin, kaempferol, quercetin, and rutin in substantive amounts. Polysaccharides mannose, glucose, and lactose were identified by GC-MS and UHPLC-MS and supported with FT-IR analysis. The extracts obtained using ethanol, methanol, and ethyl acetate showed antibacterial capability against *E. coli* and *S. aureus,* while water-and n-hexane-based extracts displayed no antibacterial property. Promising enzyme inhibition activity against tyrosinase and acetylcholinesterase, more prominent in the ethanol and methanol extracts, was established. This study might be the first to report the enzyme inhibition activity of the *A. hendersonii* extracts. This study, thus, ascertains the medicinal potentiality of *A. hendersonii.* In addition to polyphenols, the polysaccharides are also significant components in the species and may be exploited for future applications in pharmaceuticals and food industries.

## Figures and Tables

**Table 1:** GC-MS analysis showing the identified constituents in the methanol extracts *A. hendersonii* leaf.

S/N	Retention Time (min)	Chemical Name	Formula	Content
1	6.55	2,4-Dihydroxy-2,5-dimethyl-3(2H)-furan-3-one	C_6_H_8_O_4_	0.85%
2	11.395	Pyranone	C_7_H_12_O_2_	4.76%
3	13.951	5-Hydroxymethylfurfural	C_6_H_6_O_3_	3.90%
4	26.433	L-Glucose	C_6_H_12_O_6_	0.87%
5	26.764	D-Mannose	C_6_H_12_O_6_	1.47%
6	27.224	3-O-Methyl-d-glucose	C_7_H_14_O_6_	1.80%
7	28.024	β-Lactose	C_12_H_22_O_11_	5.39%
8	30.825	Palmitic acid	C_16_H_32_O_2_	5.82%
9	34.778	Oleic Acid	C_18_H_34_O_2_	0.88%
10	37.784	Oleamide	C_18_H_35_NO	1.52%
11	39.464	α-Monoolein	C_21_H_40_O_4_	0.54%
12	39.876	Fenpropathrin	C_22_H_23_NO_3_	8.66%
13	40.195	cis-Vaccenic acid	C_18_H_34_O_2_	0.11%
14	40.677	12-Methyl-E,E-2,13-octadecadien-1-ol	C_19_H_36_O	0.48%
15	42.966	β-Monoolein	C_21_H_40_O_4_	1.76%
16	44.172	Trielaidin	C_57_H_104_O_6_	0.58%
17	53.641	Ethyl iso-allocholate	C_26_H_44_O_5_	1.40%

**Table 2:** UHPLC-MS analysis of *A. hendersonii* methanol leaf extract.

S/N	RT (min)	Ion Mode	Compound	Formula	m/z	A:B
1	0.585	Neg	Mannose 6-phosphate	C_6_H_13_O_9_P	259.02	182,036.5
2	0.615	Pos	L-Fucose	C_6_H_12_O_5_	129.06	26,341.74
3	0.630	Pos	D-Glucose	C_6_H_12_O_6_	145.05	1,593,856
4	1.371	Neg	Galactose-beta-1,4-xylose	C_11_H_20_O_10_	311.10	39,867.93
5	2.426	Neg	1-O-Caffeoylglucose	C_15_H_18_O_9_	683.18	5760.163
6	2.669	Pos	Quercetin-3’-glucuronide	C_21_H_20_O_13_	481.10	167,811.6
7	2.692	Pos	Epigallocatechin gallate	C_22_H_18_O_11_	423.07	60,258.74
8	2.767	Pos	Caffeic Acid	C_9_H_8_O_4_	145.03	1,193,151
9	2.798	Pos	2-O-p-Coumaroyltartronic acid	C_12_H_10_O_7_	231.03	13,574.3
10	2.938	Pos	Isorhamnetin	C_16_H_12_O_7_	317.07	37,021.23
11	3.106	Pos	Catechin 5-glucoside	C_21_H_24_O_11_	453.14	358,651.9
12	3.217	Neg	3-O-Feruloylquinic acid	C_17_H_20_O_9_	367.10	2,787,314
13	3.267	Neg	Genistein 7-O-(2-p-coumaroylglucoside)	C_30_H_26_O_12_	577.135	6,182,057
14	3.314	Pos	Quercetin 3-galactoside	C_21_H_20_O_12_	465.10	5,091,807
15	3.338	Pos	Apigenin	C_15_H_10_O_5_	271.06	434,868.8
16	3.347	Neg	Gallic acid	C_7_H_6_O_5_	151.00	26,440.88
17	3.430	Neg	Rutin	C_27_H_30_O_16_	591.14	154,632.5
18	3.460	Pos	Quercetin	C_15_H_10_O_7_	303.05	727,312.6
19	3.630	Pos	Salicylic acid	C_7_H_6_O_3_	277.07	346,041.2
20	3.763	Neg	Epicatechin	C_15_H_14_O_6_	271.06	8479.978
21	3.783	Pos	D-Xylose	C_5_H_10_O_5_	115.04	66,500.13
22	4.067	Neg	Kaempferol	C_15_H_10_O_6_	285.04	223,062.7
23	5.404	Neg	UDP-L-rhamnose	C_15_H_24_N_2_O_16_P_2_	531.04	32,827.37
24	5.943	Neg	Apigenin 7-sulfate	C_15_H_10_O_8_S	395.01	28,169.9
25	6.931	Pos	(±)-Naringenin	C_15_H_12_O_5_	273.08	1605.806
26	10.142	Neg	Myricetin	C_15_H_10_O_8_	317.03	21,840.85
27	10.378	Neg	Luteolin	C_15_H_10_O_6_	267.03	31,687.81
28	10.996	Neg	Vanillic acid 4-sulfate	C_8_H_8_O_7_S	494.99	94,126.55
29	12.060	Neg	UDP-D-galactose(2-)	C_15_H_22_N_2_O_17_P_2-2_	609.04	122,666.8
30	12.866	Neg	Fructose 6-phosphate	C_6_H_13_O_9_P	241.01	246,177.8
31	12.941	Neg	Caffeic acid 3-sulfate	C_9_H_8_O_7_S	305.00	262,977.9
32	13.789	Neg	Luteolin 7-sulfate	C_15_H_10_O_9_S	365.00	496,272

RT: Retention time; A:B: Mean Relative content of three replicated samples.

**Table 3:** HPLC analysis of some common flavonoids constituents in *A. hendersonii* leaf extracts obtained using various solvents.

Extracts	Quercetin (µg/g)	Kaempferol (µg/g)	Rutin (µg/g)	Apigenin (µg/g)
Water	12.56 ± 0.03 ^b^	10.50 ± 0.14 ^b^	58.90 ± 0.17 ^b^	23.49 ± 0.05 ^b^
Ethanol	167.34 ± 2.59 ^e^	105.28 ± 0.12 ^d^	147.15 ± 0.10 ^d^	46.06 ± 0.03 ^d^
Ethyl acetate	43.79 ± 0.06 ^c^	30.69 ± 0.04 ^c^	78.16 ± 1.86 ^c^	29.50 ± 0.54 ^c^
n-Hexane	6.62 ± 0.21 ^a^	4.47 ± 0.21 ^a^	0.69 ± 0.06 ^a^	5.46 ± 0.03 ^a^
Methanol	113.49 ± 0.17 ^d^	127.04 ± 0.17 ^e^	171.57 ± 0.67 ^e^	58.45 ± 0.21 ^e^

Mean ± standard error of means. Means on the same column with different letters are statistically different (*p* < 0.05).

**Table 4:** Pearson correlation coefficient of the *A. hendersonii* bioactive constituents and antioxidant capacities.

Antioxidant Capacity/Bioactive Constituents	FRAP	DPPH	ABTS
Total phenolic content	0.997 ***	0.922 *	0.874
Total Flavonoid content	0.677	0.790	0.674
Total Tannin content	0.701	0.676	0.901 *
Polysaccharide content	0.971 **	0.924 *	0.795

***^,^ **^,^ * Significant correlation (*p* < 0.001, <0.01, <0.05, respectively).

**Table 5:** Antibacterial effect of various leaf extracts of *A. hendersonii* against *E. coli* and *S. aureus*.

	Zone of Inhibition Against *E. Coli* (mm)		
**Solvents**	**Extracts**	**Negative Control**	**Positive Control**
Ethanol	10.33 ± 0.32	7.75 ± 0.30	12.15 ± 0.06
Ethyl acetate	9.15 ± 0.51	7.02 ± 0.12	
n-Hexane	-	-	
Methanol	10.15 ± 0.60	6.81 ± 0.07	
Water	-	-	
	**Zone of Inhibition Against *S. Aureus* (mm)**		
Ethanol	10.96 ± 0.15 ^b^	8.48 ± 0.10	11.10 ± 0.13
Ethyl acetate	8.22 ± 0.19 ^a^	6.94 ± 0.05	
n-Hexane	-	-	
Methanol	8.90 ± 0.06 ^a^	7.02 ± 0.37	
Water	-	-	

Mean ± standard error of means. Means on the same column with different letters are statistically different (*p* < 0.05). ‘-’ not detected.

**Table 6:** Enzyme inhibition percentage of various *A. hendersonii* leaf extracts.

Extracts	Tyrosinase Inhibition (%)	AChE (%)
Water	32.10 ± 0.41 ^c^	24.38 ± 0.34 ^b^
Ethanol	62.30 ± 1.11 ^a^	30.65 ± 0.98 ^a^
Ethyl acetate	2.42 ± 0.10 ^e^	15.95 ± 0.72 ^c^
Hexane	5.34 ± 0.73 ^d^	8.27 ± 0.85 ^d^
Methanol	55.97 ± 0.89 ^b^	28.60 ± 0.04 ^a^

Mean ± standard error of means. Means in the same column with different letters are statistically different (*p* < 0.05).

## Data Availability

Data is contained within the article and Supplementary Material.

## Supplementary Materials

The following supporting information can be downloaded at: https://www.mdpi.com/article/10.3390/plants11151964/s1, Figure S1: UV-visible spectrophotometer analysis; Figure S2: GC-MS absorption spectrum; Figure S3: UHPLC-MS spectra of the identified constituents (neg. ion mode); Figure S4: Antibacterial effects of ethanol extract of *A. hendersonii* against *Staphylococcus aureus* (A) and *Escherichia coli* (B).
