# Supplementary material for: Phytochemical Composition, Antioxidant, Antibacterial, and Enzyme Inhibitory Activities of Various Organic Extracts from Apocynum hendersonii (Hook.f.) Woodson
Source: Plants (Basel). 2022 Jul 28;11(15):1964. doi: 10.3390/plants11151964 (PMC9370764; doi:10.3390/plants11151964)
Supplement: Supplementary file 1 [file plants-11-01964-s001.zip › plants-1812088-supplementary.pdf]

plants

SUPPLEMENTARY FILES

UHPLC-MS Analysis

The ACQUITY UHPLC system (Waters Corpo MA, Milford, USA) and Triple TOF 5600 System (AB SCIEX, Framingham, MA, USA) was used for the analysis with an UPLC BEH C18 column (1.7 µm, 2.1 × 100 mm) in both positive and negative ion modes. The binary gradient elution system were 0.1 % formic acid in water, v/v (A) and 0.1 % formic acid in acetonitrile, v/v (B) and separation done under the gradient: 0 min, 5% B; 2min, 20% B; 4min, 25% B; 9min, 60% B; 14min, 100% B; 18min, 100% B; 18.1 min, 5% B and 19.5min, 5%B. The flow rate was 0.4 mL/min and column temperature of 45 ℃. The samples temperature was maintained at 4℃ with an injection volume of 50 µL. Full scan mode (m/z ranges from 70 to 1000) along with IDA mode were used for data acquisition. The parameters were: Ion spray voltage, 5500 V (+) and 4500 V (−); declustering potential, 100 V (+) and 100 V (−); collision energy, 10 eV (+) and −10 eV (−); ion source temperature, 550 °C (+) and 550 °C (−); interface heater temperature, 550 °C (+) and 600 °C (−) and curtain gas of 35 PSI. Ranges of m/z and collision energy for IDA analysis were put at 25 - 1000 and 30 eV respectively.

The acquired LC/MS raw data were analysed by the progqenesis QI software (Waters Corporation, USA). Standard internal detection parameters were deselected for peak RT alignment and isotopic peaks excluded for analysis. Noise elimination level and minimum intensity were set at 10.00 and 15 % of base peak intensity respectively. The matrix obtained was further reduced by eliminating any peaks with missing value (ion intensity = 0) realized in more than 60 % samples. The internal standard was used for data QC (reproducibility). 0.3 mg/ml 2-Chloro-L-phenylalanine (CAS: 103616-89-3) in Methanol solution was use as reference substance. The date analysis was based on peak area normalization.

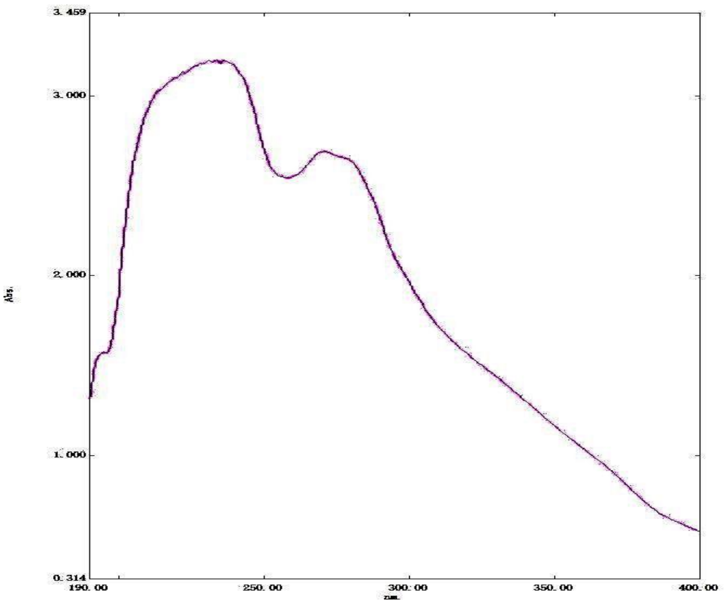

Figure S1. UV-visible spectrophotometer analysis.

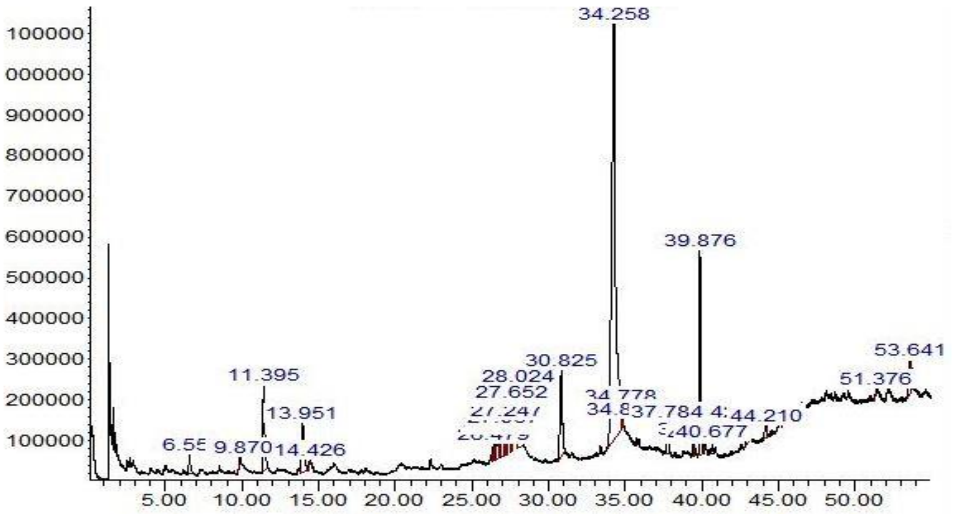

Figure S2. GC-MS absorption spectrum.

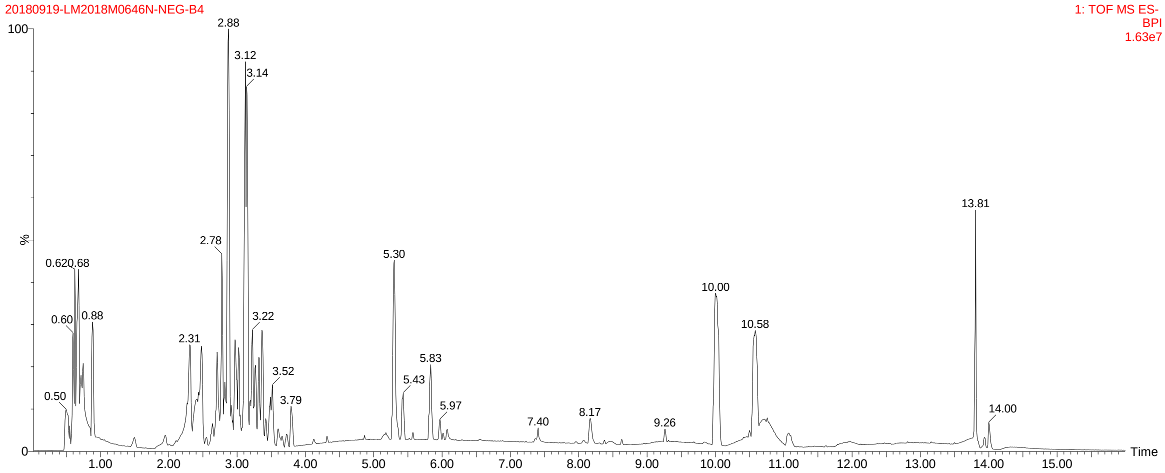

Figure S3. UHPLC-MS spectra of the identified constituents (neg. ion mode).

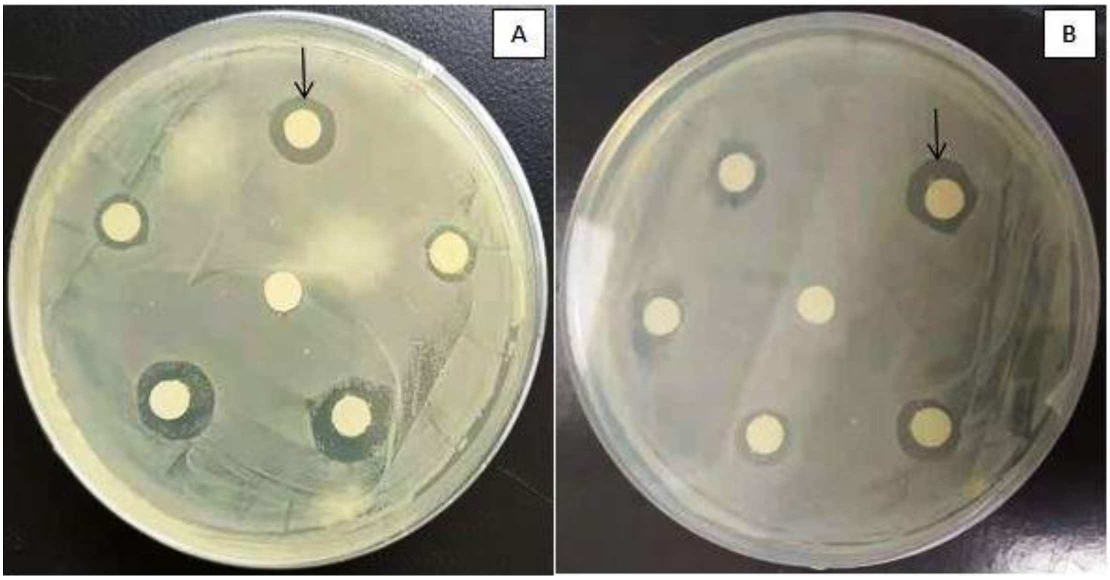

Figure S4. Antibacterial effects of ethanol extract of *A. hendorsonii*: (A) *Staphylococcus aureus*; (B) *Escherichia coli*.
